# TreeDyn: towards dynamic graphics and annotations for analyses of trees

**DOI:** 10.1186/1471-2105-7-439

**Published:** 2006-10-10

**Authors:** François Chevenet, Christine Brun, Anne-Laure Bañuls, Bernard Jacq, Richard Christen

**Affiliations:** 1 Laboratoire de Génétique et Evolution des Maladies Infectieuses, UMR CNRS/IRD 2724, IRD, 911 avenue Agropolis, BP 64501, 34394 Montpellier Cedex 5, France; 2 Institut de Biologie du Développement de Marseille-Luminy, CNRS UMR 6216, Parc Scientifique et Technologique de Luminy, Case 907, 13288 Marseille Cedex 9, France; 3 Laboratoire de Biologie Virtuelle, CNRS UMR 6543, Université de Nice Sophia Antipolis, Centre de Biochimie, Campus Valrose, 06108 Nice, France

## Abstract

**Background:**

Analyses of biomolecules for biodiversity, phylogeny or structure/function studies often use graphical tree representations. Many powerful tree editors are now available, but existing tree visualization tools make little use of meta-information related to the entities under study such as taxonomic descriptions or gene functions that can hardly be encoded within the tree itself (if using popular tree formats). Consequently, a tedious manual analysis and post-processing of the tree graphics are required if one needs to use external information for displaying or investigating trees.

**Results:**

We have developed TreeDyn, a tool using annotations and dynamic graphical methods for editing and analyzing multiple trees. The main features of TreeDyn are 1) the management of multiple windows and multiple trees per window, 2) the export of graphics to several standard file formats with or without HTML encapsulation and a new format called TGF, which enables saving and restoring graphical analysis, 3) the projection of texts or symbols facing leaf labels or linked to nodes, through manual pasting or by using annotation files, 4) the highlight of graphical elements after querying leaf labels (or annotations) or by selection of graphical elements and information extraction, 5) the highlight of targeted trees according to a source tree browsed by the user, 6) powerful scripts for automating repetitive graphical tasks, 7) a command line interpreter enabling the use of TreeDyn through CGI scripts for online building of trees, 8) the inclusion of a library of packages dedicated to specific research fields involving trees.

**Conclusion:**

TreeDyn is a tree visualization and annotation tool which includes tools for tree manipulation and annotation and uses meta-information through dynamic graphical operators or scripting to help analyses and annotations of single trees or tree collections.

## Background

Graphical management of trees requires processing and information visualization methods allowing the user to deal with single large trees or multiple connected trees. Although solutions have been proposed for the management of single and large trees [1-5], comparisons among trees [6,7], and annotations of trees [8-10], an integrated tool for the graphical management of annotations and comparisons of multiple trees is not yet available (see Discussion). Presently, there are real needs to explore, compare, display and interpret trees using information not directly contained in these trees, such as taxonomy, geography, life history traits or even ontologies [11-14]. TreeDyn aims at filling these needs. TreeDyn presently manages multiple windows, multiple trees per window, as well as related information. Meta-information may be useful for investigating a single large tree or a collection of trees. Instead of using only information located in the tree file itself (in an extended newick format, see for example [9]), TreeDyn also uses associated annotation files. TreeDyn uses a 2D Euclidean space representation to efficiently organize tree items without superposition (see Discussion).

## Implementation

TreeDyn is implemented in Tcl/Tk [15,16]. It is based on the ActiveTcl distribution which contains several Tcl/Tk extensions such as Itcl/Itk, Iwidgets, TkTable and Img. TreeDyn is a stand-alone application distributed for OSX, Linux and Windows platforms without previous installation of Tcl/Tk. Since TreeDyn is under active development, new tools will become available. Automatic TreeDyn updates ensure users to work with the latest TreeDyn version without having to visit treedyn.org, check for updates, download and install again.

## Results

### Importing exporting

Single or multiple trees can be imported from nexus and newick formats. TreeDyn allows trees to be printed or exported to several standard file formats (8 classical graphic formats, Postscript, SVG and HTML) or to a specific format called the "TreeDyn Graphic File" (TGF), which enables saving and restoring graphics. The HTML export function creates a bitmap screenshot within HTML encapsulation that may include annotations and active links associated with leaves (for example EMBL/GenBank entries). This format should facilitate the electronic publication of trees, with colors and contextual information.

### Editing

Dynamic graphics methods include two important properties: the direct manipulations of graphical elements on screen and the virtually instantaneous change of these elements [17]. In TreeDyn, tools for tree editing are available as "tool to graphical items" and "graphical items to tool" interaction modes. Using the" tool to graphical items" mode, dynamic tools can be selected and applied "on the fly" to trees, nodes, leaves, annotations, etc. For instance, the user first activates the "swap" tool and then brushes a tree for swapping. Conversely, in the "graphical items to tool" interaction mode, a graphical item is first selected within a tree and several tools can then be applied through contextual menus. For instance, a sub-tree is selected and various operations are applied onto it via its contextual menu.

Tools are represented by icons and organized into toolboxes. Two types of toolboxes are available: a default one integrating basic tools only and a toolbox dedicated to experts containing every available TreeDyn tool. Finally, a toolbox editor enables the user to build dedicated toolboxes by the selection, coloring and ordering of tools. Tools dedicated to tree manipulations allow operations such as translating trees on the canvas, zooming and navigating using global/local views, re-rooting and swapping. Leaf or subtree colors, fonts and lines are adjustable. Shrinking, collapsing, extraction of sub-trees and deletion/copy/insertion of leaves or sub-trees are also possible. Finally, one can switch among rectangular, internal or external circular tree configurations with or without proportional branch lengths.

TreeDyn enables the management of collections of trees. Multiple newick strings can be loaded as a single file and the corresponding trees displayed as a single document. It is then possible at once to resize all of the trees, organize them into rows and columns, switch the collection to a new configuration (rectangular, circular, etc.), display or hide leaf labels as well as graphical variables for the entire collection (font, foreground or background colors). It is still possible to manage each tree individually.

### Projection

**Figure 1:**
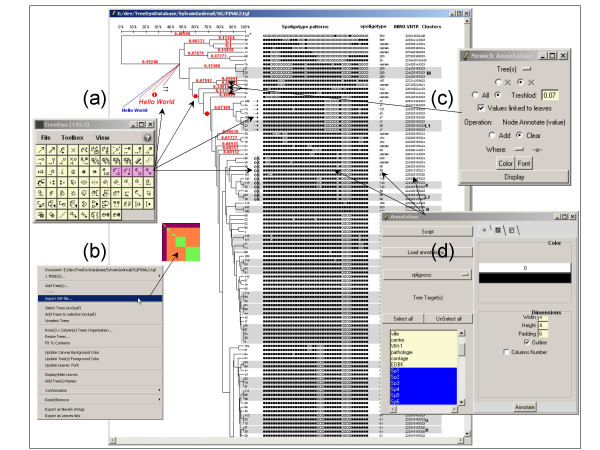
**Screen shot of a TreeDyn session using Projection functionalities**. TreeDyn enables "on the fly" annotation of nodes or leaf labels using the ToolBox **(a)**. The user selects a tool, for instance "Annotate Subtree, symbol", selects a symbol and a color and then annotates tree elements (see corresponding arrows). Bitmaps may be also imported and linked to trees (in this example a pattern visualization of the tree using the TreePAT package of TreeDyn) **(b)**. The "Newick annotation panel" **(c) **uses the information stored within the newick string such as branch lengths, bootstrap values or taxonomic levels (before or after the ":" character). The Annotation panel **(d) **enables loading and posting of annotations as graphical elements, facing tree leaves. In this example, a list of binary variables (Sp1, Sp2, etc.) is displayed as a symbol matrix where black and white dots represent the values 1 and 0 respectively in this example.

Projection is the process of posting texts, images or symbols facing a tree's elements (trees, sub-trees, nodes or leaves). Projection is implemented in three different manners. The simplest one is a manual pasting of annotations to a tree's elements. For instance, the user selects a symbol, or enters a text, and pastes it facing leaves, or as an annotation associated to a selected tree's elements (figure 1a,b). The second method is the posting of standard self-contained annotations such as branch lengths or bootstrap values (figure 1c). The third and more powerful method uses annotation files to post values facing leaf labels, possibly as a symbol-matrix for pattern visualization (figure 1d).

Annotations files use leaf labels as keys to address lists of key/values pairs. An annotation file is a simple text file containing one record per line. Each record begins with the name (label) of a leaf in a tree followed by a list of key/value(s) pairs. Annotation files can be generated by TreeDyn from tabulated ASCII files (such as generated by spreadsheets) or by using specific online tools such as GOToolbox [18].

Since TreeDyn permits the linkage of annotations to a tree's elements, the posted annotations are moved accordingly during a tree manipulation.

### Identification

**Figure 2:**
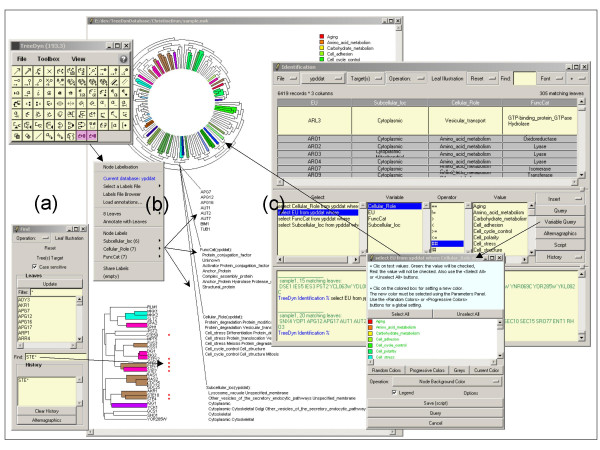
**The Identification operator, localization and labelization**. The "Find panel" **(a) **first allows selection of a highlighting operation: symbol or text annotation but also foreground or background colors, shrinking sub-tree and so on. Then the user either selects a leaf label from a list or enters a string pattern. In this example the pattern "STE*" highlights leaf labels starting with "STE" (red symbol facing leaf labels). Labelization consists in browsing a tree, selecting sub-trees and posting annotations associated to its leaves **(b)**. Localization operates in the opposite direction; it consists in querying annotations associated to leaves and highlighting tree's elements. The Identification panel **(c) **enables localization and multi-localization (localization on tree collections), it includes a SQL like interpreter. For instance, a "protein tree" is automatically colored according to 'Cellular role' annotations from Yeast Proteome Database [19].

Identification allows querying between tree elements and contents of leaf labels or annotation files. Highlighting clusters of leaves according to a specific string or pattern found in the leaf labels is possible (figure 2a). But identification is more powerful when annotation files are used. A first process called "labelization" consists in posting associated information to selected tree elements. For instance, one may select a sub-tree and the relevant annotations associated to its leaves and post these annotations as multiple and moveable new graphical text items linked to the sub-tree by connectors (figure 2b). A second process called "localization" results in highlighting every tree element associated to a certain annotation based on a SQL-like search of the annotation file. For instance, knowing a tree on the one hand and, on the other hand an annotation file containing for some leaf labels a list of variables/values pairs, the query "select Leaves from Annotations where VariableX == ValueX1" returns a list of leaf labels found in the tree and matching the "ValueX1" value for the variable "VariableX". This list is then used by a highlighting operation which updates the tree aspect (*e.g*. "sub-tree background color to red"): figure 2c, see also [19,20]. The SQL like interpreter integrated into TreeDyn enables queries on leaf label annotations using operators working on multi-valued variables ("##": contains, "!#": does not contain) with or without patterns on values (following standard rules for string pattern matching) and AND/OR connectors.

TreeDyn enables simultaneous localizations on multiple trees, either by querying leaf labels using patterns or by querying annotation files as described above. For instance, the view of a tree collection may be simplified by shrinking any sub-trees containing a particular string pattern within the leaf labels. Similarly, modifying the foreground (background) color of sub-trees carrying leaves having identical values for a given variable (*e.g*. in a phylogenetic study of host-parasite co-speciation, a host tree is colored according to parasites) is possible. Each of these operations helps interpreting sets of trees, facilitating the detection of similarities or differences between trees.

### Reflection

**Figure 3:**
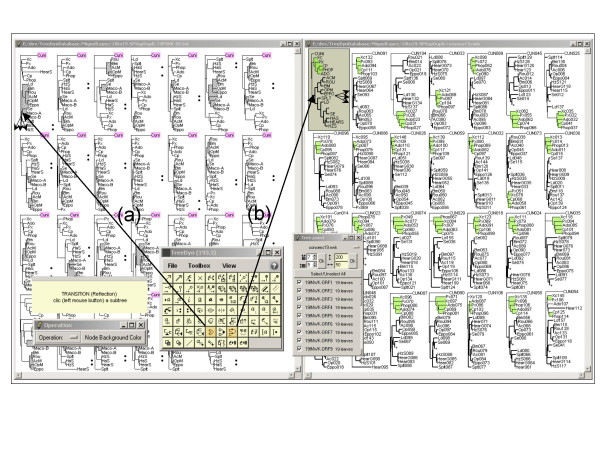
**The Reflection operator for tree collection analyses**. Screenshot illustrating reflection used in the study of 27 gene trees from 19 virus species of the *Baculoviridae *family [38]. Reflection enables the browsing of a source tree and results in highlighting targeted trees. In example **(a) **the Reflection tool using a strict identity of leaf labels between trees is activated. It enables the selection of the sub-tree {Ld, Bm, Rou, AcM, OpM, Eppo, Se, Maco-A and Maco-B} and allows its highlighting (grey sub-tree background) and every potentially sub-trees of others tree of the collection. Any tree of the collection may be used as a source tree and different highlighting operations are available. Here a symbol insertion facing "HaS" and "HzS" leaf labels and the update of the background for the outgroup"Cuni". In example **(b)**, The Reflection tool using different leaf label sets is activated. The reflection operates from a consensus tree (tree with a grey background) using species names as leaf labels towards a set of trees using genes names as leaf labels. The corresponding annotations file integrates records putting in regards species labels from the consensus tree to genes labels from the others trees (*e.g*. "ADO reflection {Ado001 Ado002 Ado003 ...}"). With such an annotations file, the reflection operates from the consensus tree (the source tree) to the other trees, and results in highlighting sub-trees background, shrinking and line aspect modifications. For a reflection between genes trees, the annotation file may integrate records linking leaf labels from different trees, as described in the text (for example "Ado001 reflection ADO", "Ado002 reflection ADO"...).

The reflection operator allows the comparison of trees carrying identical or different but related leaf labels. This operator allows the highlighting of targeted trees according to a source tree browsed by the user. Two methods are proposed for this operation. The first method relies on a strict identity of leaf labels between trees (figure 3a). Selection of a sub-tree from a tree allows highlighting every identical leaf in others trees (and potentially sub-trees, depending of their topologies and the highlighting operation chosen). The second method deals with trees bearing different leaf labels (for example different genes families, see figure 3b). In this case, the reflection operator requires an annotation file in which the connections between leaf labels in the different trees are described by value(s) of variable(s). For instance, if one wants the selection of leaf "a1" from the source tree "a" to highlight leaves "b1 b2 b3" of the tree "b", the corresponding annotation file must include records linking the leaf labels from tree "a" to that of tree "b", for example in the form "a1 string {b1 b2 b3}" where "string" is any variable's name. Reflection works with any kind of variable. For instance, the user selects a source sub-tree, and then chooses a variable from an annotations file (*e.g*. Country) and a particular value for this variable (*e.g*. Country X). TreeDyn then highlights every targeted tree for corresponding leaf labels (and possibly potential sub-trees) matching the value "X" for the "Country" variable, allowing powerful comparisons between trees.

### Scripting

A TreeDyn script file (ASCII format) containing a list of instructions is a way of saving graphical analyses and avoids repetitive tasks. A scripting package includes a language dedicated to the treatment of trees and annotations. This language is based on the description of aliases between a master interpreter which is running TreeDyn and a slave interpreter waiting for user instructions. Every operation available from the TreeDyn interface is scriptable.

Scripts are loadable either through the TreeDyn interface or can be run from the command-line. For example, the command "treedyn -tree *treeFile *-label *labelFile *-script *scriptFile *-out *outFile*" applies a graphical treatment as described in *scriptFile *on a tree (*treeFile*) using annotations stored in *labelFile *returning Postscript and TGF outputs (outFile.ps and outFile.tgf). Such functionality enables TreeDyn to be linked to HTTP servers through CGI scripts as illustrated by the Prodistin Web Site [21], which uses TreeDyn for tree representations.

### TreeDyn Packages

TreeDyn Package is an open library of modules dedicated to specific tree graphical management tasks. We present four examples of such packages: TreeBASEinterf, TreeIG, TreePAT and TreeXY.

**Figure 4:**
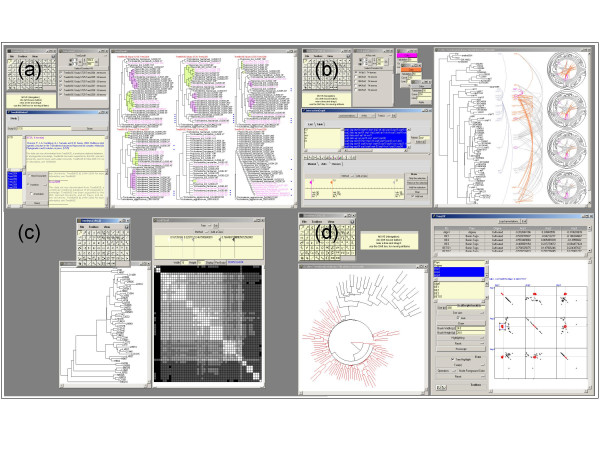
**Screen shots of TreeDyn packages**. Knowing an ID Study (*e.g*. S725) from TreeBASE, the TreeBASEinterf module **(a) **allows the fetching of selected tree(s) and their insertion in a TreeDyn document. The TreeIG package **(b) **allows the drawing of arcs facing the selected leaf labels. Graphical variables of arcs (color, curvation, tabulation) follow a user's definition possibly based on the distances between the leaves. The TreePAT package **(c) **enables the computation and the coloring of the distance matrix between leaves based on branch lengths. The TreeXY **(d) **package allows a dynamic linkage between trees and scatterplot matrices.

*TreeBASEinterf *(figure 4a). TreeBASE is a relational database designed to manage and explore information on phylogenetic relationships [22-25]. The database is designed to allow the retrieval of trees and data from different studies and can be explored interactively. Knowing keywords (taxa, author names, etc.), TreeBASE web interface allows retrieval of studies (published research papers) and their related information (methodology, datasets, trees). Trees can be displayed or downloaded and viewed with TreeDyn. Knowing ID studies from TreeBASE, TreeBASEinterf allows fetching selected trees and their treatment with the TreeDyn tools, without any constraint due to security policies such as encountered when using applet technology (saving, printing). Downloaded trees can be displayed as a collection in new TreeDyn documents or inserted in already existing documents for comparison with trees built by the user.

*TreeIG *(figure 4b) allows the drawing of arcs between leaves of a tree. It may be used to display additional relationships existing between leaves which are not represented by the tree itself, such as interactions between proteins. TreeIG uses annotation files storing these relationships as variables. Knowing a user selection of leaves, through the selection of a subtree or through the selection from a list (an extended selection is available, with or without pattern matching), arcs are drawn according to four graphical variables: curvature, line-width, color and tabulation (a user specification).

*TreePAT *(figure 4c) allows the representation of a tree as a pattern visualization matrix. A pair-wise distance matrix is computed according to the distances of the leaves in the tree (sums of branch lengths). Some classes are then defined as ranges of distances from 0 (a leaf to itself) to the diameter of the tree. Finally, a color is associated to each class resulting in the distance matrix being colored accordingly. Leaves within a given distance class appear with their associated color as squares more or less well structured along the diagonal of the matrix.

*TreeXY *(figure 4d) enables a dynamic linkage between trees and scatterplot matrices. For instance, the module may help in the co-analysis of a given set of species represented on the one hand as a phylogenetic tree based on molecular data, and on the other hand, as a scatterplot matrix of factorial maps from a multivariate analysis using geographic data. A toolbox allows mouse-driven selections of sub-sets of species, from the tree or from the scatterplot representation, and their highlighting, respectively on the scatterplot and on the tree. Different modes of interaction allow different highlighting operations and to keep/undo results following several selections.

## Discussion

### Layout

Many tools for visualising phylogenetic trees already exist; they first differ in their layout, i.e. 2D or 3D and using Euclidean or hyperbolic representation. Most popular tools such as Treeview [1] and ATV [9] lay out trees in a two dimensional Euclidean space and are useful for visualising trees of up to a few hundred nodes; PoInTree makes uses of polar coordinates [26]. Tools, such as Hypertree [3], have increased the number of visualisable nodes using 2D hyperbolic space providing a "focus+context" view, where a subset of the data can be viewed at a higher resolution with the remaining contextual data still being in view. In hyperbolic space (as opposed to Euclidean space), circumference and area increase exponentially instead of geometrically. It enables allocation of space for every node independent of the total number of nodes in the tree, which can be projected into a finite volume of Euclidean space for a "focus+context" view. By bringing different parts of a tree to the magnified central region, the user can examine every part of the tree in detail while retaining a sense of the context. Hypertree allows visualization of up to a thousand nodes [3]. In order to handle an order of magnitude more nodes, one strategy is to not visualise the whole tree but instead to display a representative part of it as implemented in SpaceTree and TreeWiz [4,27]. Visualization using virtual reality has also been reported as a potential approach to the problem, but this requires a special virtual reality chamber [28,29]. More recently, hyperbolic representation made use of 3D coordinates [5,30] making possible to interactively visualize the entirety of trees with several hundred thousand nodes on a desktop computer. Hyperbolic representations are fine for global visualizations of large datasets, but suffer from unresolved problems of leaf label and annotation management to avoid superposition; besides the main aim of TreeDyn is to produce figures for publication (printed or browsing); it was therefore designed to use a standard 2D Euclidean space, with every alternate layout being feasible (phylogram, rectangular or slanted cladogram, radial view, circular inside and outside, with or without proportional branch lengths). Using the combination of global and local navigators, trees of up to 15 000 leaves have been successfully viewed with TreeDyn.

### Aspect

Once a tree represented in a 2D Euclidean space, easy changes of aspect of edges as well as leaf labels are required (line width and aspect, font, size of labels...). Most popular tree editors allow such operation either for the entire tree or for a selection of items. Both can be done with TreeDyn, which includes many more alternate options than any other tree editor. Also, apart from manual selections and changes, TreeDyn allows extensive scripting to be used. TreeGraph [31] assists in producing complex ready-to-publish figures of phylogenetic trees through scripting, but with much less possibilities. PAL (Phylogenetic Analysis Library, [32]) would be an alternate possibility, but for the moment it is not implemented through a visual interface and has also less functionalities.

### Export trees

Saving can be done toward almost any image format, post-script, SVG and as "live" encapsulated html file. To our knowledge, no other editor is capable to do so, excepting TreeGraph [31] which also exports to SVG. In addition, TreeDyn provides the user with the specific TGF format enabling the saving and restoring of analyses.

### Comparing trees

Since there are many methods for building trees, and also many sources of information for building a tree from the same objects (genes for a species tree for example), it is often desirable to summarize or compare a set of phylogenetic trees [33]. Several approaches are now available from the "simple" consensus tree [34] to the visualization of a "tree space" using multi-dimensional scaling based on a tree-to-tree distance matrix (Tree Set, [13,35]) or to systems allowing detailed structural comparisons between trees of up to 100,000 nodes (TreeJuxtaposer, [6]). One may however wish not to compare a set of trees in their entirety, but only for a subset of leaves (*e.g*. a clade) of interest. TreeDyn offers a solution to manage multiple trees, using leaf labels as unique key to record lists of variables/values pairs, independently of the tree topologies. This information is used by graphical operators that allow highlighting, annotating or shrinking nodes or leaves among the set of trees, therefore providing an instant representation of congruence or divergence. In this respect, TreeDyn is more powerful than the above mentioned tools since it allows linking and highlighting leaves that have a different content through the use of an annotation file.

### Annotations

Usual tree description formats (newick [36] or nexus[37]) used by most phylogenetic software or tree-drawing tools do not allow the easy inclusion of additional information (except support value and/or branch length). As a consequence, additional information needs to be manually added to the tree with the help of a graphic editor. This operation can often be inferred from subtle inhomogeneous arrangements in the final figures. An attempt to arrange and format these elements is very time consuming and may involve human errors. TreeGraph [31] extends the usual parenthetical tree notation (Newick and similar formats) to include much more information for each branch or node, such as different support value types, text and graphical labels. Using its command line editor, it is then possible to add annotations, change label's fonts and modify the tree structure to produce a publication ready figure. TreeDyn offers an improved solution to manage such meta-information, by using external annotation files in the form of key-values couples. The annotation procedure of TreeDyn is easier (a command can be tested within the tree editor and its effect can be instantaneously visualized), more powerful as it may use large, easy to build annotation files. Also, these procedures can be applied to a series of trees. Finally, by keeping annotations external to the tree description itself, a single tree can be annotated with different annotation files for different contexts.

## Conclusion

Tree analyses often need an alternate focusing between complex tree graphical structures and information related to the entities under study. TreeDyn offers a solution to manage, on the one hand, multiple trees, and on the other hand, meta-information. TreeDyn offers to link unique leaf labels to lists of key/values pairs, independently of the tree topologies, remaining fully compatible with the basic newick format. These relationships are used by graphical operators allowing a Human-Computer interaction ranging from manual (user driven) to "all automatic" (computer driven) processes: from annotations to trees, from trees to annotations, from trees to trees through annotations. The scripting capability is an improvement towards the automation of graphical "error free" treatments and its use with the Treedyn command line enables TreeDyn to be linked to HTTP servers through CGI scripts. TreeDyn is under active development, and suggestions for improvements are welcome (as for example import of specific formats). As TreeDyn is under the GPL licence, any development by a third party is also welcome. Full documentation as well as tutorials are available on the TreeDyn web site [39].

## Availability and requirements

• **Project name: **TreeDyn

• **Project home page: **

• **Operating systems: **MacOSX, Linux, Windows

• **Programming language: **Tcl/Tk, ActiveTcl 8.4.3

• **Other requirements: **none

• **License: **GPL

• **Any restrictions to use by non-academics: **none

## Authors' contributions

FC designed and led the project, developed the algorithms, prototypes, coded and integrated the complete package. CB, ALB, BJ and RC provided biological insights, data and tested the software. FC and RC essentially wrote the paper with contributions of CB. RC and FC had regular stimulating discussions regarding the evolution of the software. All authors read and approved the final manuscript.

## References

[B1] Page RD (1996). TreeView: an application to display phylogenetic trees on personal computers. Comput Appl Biosci.

[B2] Perrière G, Gouy M (1996). WWW-Query: An on-line retrieval system for biological sequence banks. Biochimie.

[B3] Bingham J, Sudarsanam S (2000). Visualizing large hierarchical clusters in hyperbolic space. Bioinformatics.

[B4] Rost U, Bornberg-Bauer E (2002). TreeWiz: interactive exploration of huge trees. Bioinformatics.

[B5] Hughes T, Hyun Y, Liberles DA (2004). Visualising very large phylogenetic trees in three dimensional hyperbolic space. BMC Bioinformatics.

[B6] Munzner T, Guimbretiere F, Tasiran S, Zhang L, Zhou Y (2003). TreeJuxtaposer: Scalable Tree Comparison using Focus+Context with Guaranteed Visibility. SIGGRAPH: ACM Transactions on Graphics.

[B7] Hillis DM, TA H, K SJ (2005). Analysis and Visualization of Tree Space. Syst Biol.

[B8] Chevenet F, Bañuls AL, Barnabé C, Caraux G, Gascuel O, Sagot MF (2000). TreeDyn: un éditeur interactif d'arbres phylogénétiques. Actes des Premières Journées Ouvertes Biologie, Informatique et Mathématiques ENSAM/Montpellier.

[B9] Zmasek CM, Eddy SR (2001). ATV: display and manipulation of annotated phylogenetic trees. Bioinformatics.

[B10] Pasquier C, Girardot F, Jevardat de Fombelle K, Christen R (2004). THEA: ontology-driven analysis of microarray data. Bioinformatics.

[B11] Tao Y, Liu Y, Friedman C, Lussier YA (2004). Information visualization techniques in bioinformatics during the postgenomic era. Drug Discovery Today: BIOSILICO.

[B12] Carrizo SF (2004). Phylogenetic trees: an information visualisation perspective. Proceedings of the second conference on Asia-Pacific bioinformatics.

[B13] Amenta N, Klingner J (2002). Case Study: Visualizing Sets of Evolutionary Trees. IEEE Symposium on Information Visualization (InfoVis'02).

[B14] Lott PL, Mundry M, Sassenberg C, Lorkowski S, Fuellen G (2006). Simplifying gene trees for easier comprehension. BMC Bioinformatics.

[B15] Ousterhout JK (1994). Tcl and the Tk Toolkit.

[B16] Welch BB (2003). Practical Programming in Tcl and Tk.

[B17] Cleveland WS, McGill ME (1998). Dynamic Graphics for Statistics.

[B18] Martin D, Brun C, Remy E, Mouren P, Thieffry D, Jacq B (2004). GOToolBox, functional analysis of gene datasets based on Gene Ontology. Genome Biology.

[B19] Brun C, Chevenet F, Martin D, Wojcik J, Guénoche A, Jacq B (2003). Functional classification of proteins for the prediction of cellular function from a protein-protein interaction network. Genome Biology.

[B20] Zhong W, Sternberg PW (2006). Genome-Wide Prediction of C. elegans Genetic Interactions. Science.

[B21] Baudot A, Martin D, Mouren P, Chevenet F, Guenoche A, Jacq B, Brun C (2006). PRODISTIN web site: a tool for the functional classification of proteins from interaction networks. Bioinformatics.

[B22] Sanderson MJ, Baldwin BG, Bharathan G, Campbell CS, Ferguson D, Porter JM, Von Dohlen C, Wojciechowski MF, Donoghue MJ (1993). The growth of phylogenetic information and the need for a phylogenetic database. Syst Biol.

[B23] Sanderson MJ, Donoghue, Piel W, Eriksson T (1994). TreeBASE: a prototype database of phylogenetic analyses and an interactive tool for browsing the phylogeny of life. Amer Jour Bot.

[B24] Donoghue MJ (1994). Progress and prospects in reconstructing plant phylogeny. Ann Missouri Bot Gard.

[B25] Morell V (1996). TreeBASE: the roots of phylogeny. Science.

[B26] Marco C, Eleonora G, Luca S, Edward PS, Antonella I, Roberta B (2005). PoInTree: a polar and interactive phylogenetic tree. Genomics Proteomics Bioinformatics.

[B27] Plaisant C, Grosjean J, Bederson BB (2002). SpaceTree: supporting exploration in large node link tree, design evolution and empirical evaluation. Information Visualization. INFOVIS IEEE Symposium.

[B28] Ruths DA, Chen ES, Ellis L (2000). Arbor 3D: an interactive environment for examining phylogenetic and taxonomic trees in multiple dimensions. Bioinformatics.

[B29] Stolk B, Abdoelrahman F, Koning A, Wielinga P, Neefs JM, Stubbs A, de Bondt A, Leemans P, vdS P (2002). Mining the human genome using virtual reality. Fourth Eurographics Workshop on Parallel Graphics and Visualization: 9–10 September Blaubeuren Germany.

[B30] Munzner T (2000). Interactive Visualization of Large Graphs and Networks.

[B31] Müller J, K M (2004). TreeGraph: automated drawing of complex tree figures using an extensible tree description format. Molecular Ecology Notes.

[B32] Drummond A, Strimmer K (2001). PAL: an object-oriented programming library for molecular evolution and phylogenetics. Bioinformatics.

[B33] Day W (1985). Optimal algorithms for comparing trees with labeled leaves. Journal of Classification.

[B34] Bryant D (2003). A classification of consensus methods for phylogenetics. DIMACS Series in Discrete Mathematics and Theoretical Computer Science.

[B35] Montealegre I, St John K (2002). Visualizing Restricted Landscapes of Phylogenetic Trees. http://comet.lehman.cuny.edu/treeviz/papers/Evolu_Montealegre_20030601_061139.pdf.

[B36] Felsenstein J (1986). The Newick tree format. http://evolution.genetics.washington.edu/phylip/newicktree.html.

[B37] Maddison DR, Swofford DL, Maddison WP (1997). NEXUS: an extensible file format for systematic information. Syst Biol.

[B38] Simon O, Chevenet F, Williams T, Caballero P, Lopez-Ferber M (2005). Physical and partial genetic map of Spodoptera frugiperda nucleopolyhedrovirus (SfMNPV) genome. Virus Genes.

[B39] TreeDyn. http://www.treedyn.org.

